# A Large Cohort Study Concerning Age-Dependent Impacts of Anthropometric Variables on Spirometric Parameters in Nonsmoking Healthy Adults

**DOI:** 10.1371/journal.pone.0100733

**Published:** 2014-06-23

**Authors:** Hisamitsu Omori, Ayumi Onoue, Takahiko Katoh, Yasuhiro Ogata, Hidetoshi Kawashima, Naoki Miyao, Takao Tsuji, Kazutetsu Aoshiba, Atsushi Nagai, Kazuhiro Yamaguchi

**Affiliations:** 1 Department of Biomedical Laboratory Sciences, Kumamoto University, Kumamoto, Japan; 2 Department of Public Health Faculty of Life Sciences, Kumamoto University, Kumamoto, Japan; 3 Health Care Center, Japanese Red Cross Kumamoto, Kumamoto, Japan; 4 Internal Medicine, Nihon Koukan Hospital, Kawasaki, Japan; 5 Department of Respiratory Medicine, Tokyo Medical University Ibaraki Medical Center, Ibaraki, Japan; 6 The First Department of Medicine, Tokyo Women's Medical University, Tokyo, Japan; 7 Comprehensive and Internal Medicine, Tokyo Women's Medical University Medical Center East, Tokyo, Japan; Taipei City Hospital, Taiwan

## Abstract

**Backgrounds:**

Although height (H) has been considered the principal anthropometric variable governing lung function, the age-dependent differences in its influences on determining spirometric parameters (SPs) have not been conclusively investigated. Moreover, there has been no study centered on age-dependent effects of other anthropometric variables, including body weight (BW) and body fat mass (BFM) on SPs. In addition, the age-dependent influences of these anthropometric variables are anticipated to differ quantitatively between male and female participants.

**Methods:**

A total of 16,919 nonsmoking healthy Japanese adults (men: 6,116, women: 10,803) were partitioned into six groups stratified by gender and age at intervals of 20-years: young-, middle-, and advanced-age groups of either gender. Using a model in which a SP was described by a logarithmic additive function of age, H, BW, and BFM, we determined the partial regression coefficients of the respective anthropometric variables to predict the reference means of SPs, including FVC, FEV_1_, FEV_1_/FVC, PEF, FEF_50_, and FEF_75_, in the six groups.

**Results/Discussion:**

Although the impact of H on FVC and FEV_1_ was relatively homogeneous irrespective of gender and age, its homogeneity faded for flow parameters, particularly in the female middle- and advanced-age groups, indicating that the age-dependent contribution of H to SPs was enhanced more in women. The impact of BW on SPs differed depending on age, and this effect was also more conspicuous for female participants. H and BW generally exerted positive effects on SPs, whereas BFM had negative effects. Opposite effects of BW and BFM were observed in the female middle-age group in particular.

**Conclusions:**

The effects of anthropometric variables on spirometric parameters are highly age-dependent, particularly in women, leading to the conclusion that the assumption of age-independent, constant partial regression coefficients of anthropometric variables while predicting the reference mean of a certain spirometric parameter may result in substantial errors.

## Introduction

Overwhelming numbers (more than 120 papers) of ethnic-specific regression equations for predicting reference means and/or lower limits of normal (LLN) concerning various spirometric parameters in adults have been published over several decades [1]. Most of these equations were generated, however, by introducing age (A) and height (H) as explanatory variables. Incorporating the LMS (lambda, mu, and sigma) method with a smoothing function, Stanojevic et al. [2] recently demonstrated novel regression equations covering an entire range of age from preschool children to elderly persons in non-Hispanic white subjects. Extending the study of Stanojevic et al., the Global Lungs Initiative (GLI), an ERS Task Force, developed more global multi-ethnic regression equations with two explanatory variables, A and H, applicable across all ages from 3 to 95 years old for people of varied races [3]–[5]. The reason why most of the studies performed in this field adopted only H as the explanatory anthropometric variable may be based on the fact that not only is the lung volume modeled most appropriately by the H (besides age), but, in addition, the goal of such studies is to develop a parsimonious equation that is easily handled in clinical situations [3]. Although this consideration is appreciated from a clinical point of view, it does not imply that other anthropometric variables play no role in deciding spirometric parameters. In fact, numerous studies have been conducted to elucidate the effects of varied anthropometric variables other than H on spirometric parameters, though their impacts were not conclusively certified [6]–[19]. The common assumption in all the studies described above is that the effect of anthropometric variables, including H, body weight (BW), body mass index (BMI), or body fat mass (BFM) on a given spirometric parameter is independent of age. However, bodily shape or physique may change with aging. For instance, BW generally increases in middle age but decrease in advanced age [7]. H commonly decreases with increasing age. These facts indicate that the quantitative effect of a certain anthropometric variable on the decision of a spirometric parameter should not be taken to be constant but instead considered to change depending on age. Based on these considerations, we generated the following hypotheses in the present study: 1) spirometric parameters would be influenced by a variety of anthropometric variables, including H, BW, and BFM; 2) their effects would differ depending on age, i.e., age-specific difference in contribution of an anthropometric variable to a spirometric parameter; and 3) the extent of age-dependent contribution of an anthropometric variable would differ qualitatively and quantitatively between men and women, i.e., there are gender-specific differences in the effects of an anthropometric variable on a spirometric parameter. To test these hypotheses, we examined the relationship between anthropometric variables and spirometric parameters in six groups stratified by gender and age at intervals of 20 years. Each group consisted of thousands of healthy nonsmoking Japanese adults with a relatively narrow age span, i.e., 20 to 39 years old (young-age group), 40 to 59 years old (middle-age group) or more than 60 years old (advanced-age group). We believe that the investigation of groups with a narrow age range would be aid in extracting the age-specific contributions of anthropometric variables to spirometric parameter values.

## Methods

### Ethics Statement

All participants provided written informed consent indicating they agreed their data could be used for clinical research. The participants were asked whether they agreed with the registration of their details in the database for various research programs. Our research protocol was approved by the Human Ethics Committee of the Japanese Red Cross Kumamoto Health Care Center (registration number: 137).

### Study population

Healthy nonsmokers in the general population were sorted from those undergoing a medical checkup at the Japanese Red Cross Kumamoto Health Care Center during the two years from April 2008 to March 2010. The medical checkup included a questionnaire, test of physical strength and fitness, spirometry, chest X-ray, electrocardiogram (ECG), various blood tests, and medical examination by a physician. Nonsmokers were defined as the individuals who declared, in the questionnaire, that they never smoked before the medical checkup. Among the nonsmokers, the subjects who were reported to have no occupational history exposed to either biomass fuels or dusts and no obvious respiratory symptom, including dyspnea on exertion, nocturnal dyspnea, cough, sputum, or wheezing, were selected as the healthy nonsmoker candidates. The medical histories, as well as the results of various blood examinations, chest X-ray, and ECG, of these candidates were carefully inspected by the staff physicians. The candidates, who were confirmed to have neither cardiovascular disease nor respiratory diseases, such as lung cancer, bronchial asthma, COPD, interstitial lung disease, or infiltrative lung disease, were defined as healthy nonsmokers. The staff physicians excluded the nonsmokers who took the special medicines for asthma, COPD, and cardiovascular diseases excepting hypertension. In addition, they excluded the nonsmokers having serious systemic diseases such as malignancy in any organ, renal failure requiring dialysis, diabetes mellitus with insulin therapy, and so on. However, the nonsmokers with hypertension or hyperlipidemia were accepted as the healthy nonsmokers unless their diseases were serious. Subsequently, the respiratory specialists checked the acceptability of spirometric data and finally decided the participants eligible for the analysis. Through this process, the healthy nonsmokers with no spirometric data or incomplete and/or unacceptable traces of forced expiratory flow-volume curves were excluded. Thus, the total of 56,829 subjects over 20 years old (men: 34,160, women: 22,669) undergoing the medical checkup during the two years were assessed for eligibility and the 16,919 healthy nonsmoking adults (men: 6,116, women: 10,803) were finally enrolled for the analysis.

These participants were partitioned into three groups stratified by age at intervals of 20 years. The participants whose age was less than 39 years old were categorized as the young-age group, whereas those ranging between 40 to 59 years old were categorized as the middle-age group. The participants whose age was more than 60 years old were assigned to the advanced-age group. Among the men, the young-age, middle-age, and advanced-age groups consisted of 1,106, 3,343, and 1,667 subjects, respectively (Table 1). The number of female subjects forming the young-age, middle-age, and advanced-age groups was 1,578, 6,427, and 2,798, respectively (Table 2). These classifications were made on the ground of the preliminary results obtained for the 12 groups stratified by gender and age at intervals of 10 years, i.e. the group with age ranging from 20 to 29 years, 30 to 39 years, 40 to 49 years, 50 to 59 years, 60 to 69 years, or more than 70 years in each gender. We estimated the effects of explanatory variables, including age and anthropometric factors of height, body weight, and body fat mass, on various spirometric parameters in these 12 groups. The results showed that, in each gender, the effects of explanatory variables on spirometric parameters were qualitatively and quantitatively the same between the two successive age-groups in a first approximation (data not shown), indicating that the two successive age-groups would be united. Thus, the combination of the age-group with 20–29 years and that with 30–39 years formed the young-age group, whereas the group with 40–49 years and that with 50–59 years formed the middle-age group. Similarly, the unification of the group with 60–69 years and that with more than 70 years made up the advanced-age group. We believe that the simplification of the group classification may hasten the comprehension on the intricate results observed in this study.

**Table 1:** Demographic, anthropometric, and spirometric characteristics of male participants.

	Young-age Group (n = 1,106)	Middle-age Group (n = 3,343)	Advanced-age Group (n = 1,667)
Age (years)	35.7±2.8	49.4±5.8^*^	66.7±5.7^*,†^
Height (cm)	171.6±5.7	169.8±6.0^*^	164.7±5.9^*,†^
Body weight (kg)	70.0±11.1	69.6±10.5	63.2±8.5^*,†^
BMI (kg/m^2^)	23.8±3.5	24.1±3.2^*^	23.3 ± 2.7^*,†^
%FAT (%)	22.9 ± 5.4	22.8 ± 5.1	20.5 ± 4.7^*,†^
BFM (kg)	16.5 ± 6.3	16.2 ± 5.9	13.2 ± 4.4^*,†^
FVC (L)	4.25 ± 0.79	3.95 ± 0.74^*^	3.56 ± 0.67^*,†^
FEV_1_ (L)	3.51 ± 0.68	3.17 ± 0.60^*^	2.79 ± 0.55^*,†^
FEV_1_/FVC (%)	82.72 ± 5.75	80.50 ± 5.48^*^	78.56 ± 5.67^*,†^
PEF (L/s)	8.41 ± 1.77	8.19 ± 1.87^*^	7.53 ± 1.71^*,†^
FEF_50_ (L/s)	4.44 ± 1.34	4.00 ± 1.27^*^	3.47 ± 1.21^*,†^
FEF_75_ (L/s)	1.58 ± 0.66	1.20 ± 0.50^*^	0.90 ± 0.44^*,†^

Values are the means ± SD. BMI: body mass index. %FAT: fat percentage of body mass. BFM: body fat mass calculated from body mass (kg) multiplied by %FAT/100. ^*^: significantly different from the young-age group (at least p<0.01). ^†^: different from the middle-age group (at least p<0.01).

**Table 2:** Demographic, anthropometric, and spirometric characteristics of female participants.

	Young-age Group (n = 1,578)	Middle-age Group (n = 6,427)	Advanced-age Group (n = 2,798)
Age (years)	35.6 ± 3.0	49.7 ± 5.8^*^	66.1 ± 5.4^*,†^
Height (cm)	159.5 ± 5.2	156.9 ± 5.4^*^	152.3 ± 5.1^*,†^
Body weight (kg)	53.3 ± 8.6	54.4 ± 8.6^*^	52.1 ± 7.6^*,†^
BMI (kg/m^2^)	20.9 ± 3.2	22.1 ± 3.3^*^	22.4 ± 3.0^*,†^
%FAT (%)	26.2 ± 6.4	27.8 ± 6.2^*^	27.9 ± 5.8^*^
BFM (kg)	14.4 ± 6.1	15.6 ± 6.0^*^	14.9 ± 5.1^*,†^
FVC (L)	3.25 ± 0.59	3.12 ± 0.64^*^	2.82 ± 0.72^*,†^
FEV_1_ (L)	2.73 ± 0.46	2.53 ± 0.51^*^	2.23 ± 0.59^*,†^
FEV_1_/FVC (%)	84.27 ± 6.32	81.28 ± 5.65^*^	79.24 ± 5.47^*,†^
PEF (L/s)	6.01 ± 1.49	6.09 ± 1.56	5.65 ± 1.65^*,†^
FEF_50_ (L/s)	3.60 ± 0.96	3.31 ± 1.01^*^	2.87 ± 1.08^*,†^
FEF_75_ (L/s)	1.35 ± 0.55	0.99 ± 0.44^*^	0.75 ± 0.42^*,†^

Values are the means ± SD. Symbols are the same as denoted in Table 1. ^*^: different from the young-age group (at least p<0.03). ^†^: different from the middle-age group (at least p<0.03).

### Spirometry and measurements of anthropometric measurements

Forced expiratory pulmonary function tests were performed using an electric spirometer (DISCOM-21 FX, CHEST Co., Tokyo, Japan). Maneuvers were performed according to the standardization of lung function testing recommended by the ATS/ERS Task Force [20]. Under supervision by a skilled technician for lung function tests, the participant repeated the forced expiratory maneuver until at least three acceptable and reproducible blows were obtained. Checking the values of FVC, FEV_1_, and extrapolated volume as well as the shape of the flow-volume curve, the technician selected the best FVC blow among the three traces. The trace thus selected was inspected by a respiratory specialist, who finally decided whether the best FVC trace of the participant was acceptable for the further analysis. We adopted FVC, FEV_1_, FEV_1_/FVC, PEF, FEF_50_, and FEF_75_ as forced expiratory spirometric parameters for the subsequent analysis.

A variety of anthropometric variables, including standing height (H, cm), body weight (BW, kg), body mass index (BMI), and fat percentage of body mass (%FAT, %), were examined for all participants. Standing height was measured with a stadiometer without shoes, while the participant was weighed on a scale wearing a suite of light clothes (weight: 500 g). Blood examinations and %FAT measurements were performed under fasting conditions without taking the breakfast. %FAT was measured with a bioelectrical impedance method (BF-220, TANITA Co., Tokyo, Japan). Body fat mass (BFM, kg) was calculated by multiplying BW by (%FAT/100).

### Multivariate analysis and selection of anthropometric variables

Extending the model proposed by the GLI [3]–[5], we assumed that the effect of various explanatory variables (E_1_, E_2_, E_3_⋅⋅⋅E_n_) on the spirometric parameter (SP) was described by the multiplicative exponential function. Thus, the regression model for a certain SP assumed the following:
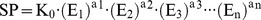
(1)where K_0_ is the constant equal to Exp(a_0_). By taking the logarithm (Ln) of both sides of eq. (1), the model is converted to the logarithmic additive function as follows:
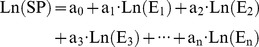
(2)


In eq. (2), a_i_ (i = 1 to n) is the partial regression coefficient of a particular explanatory variable, whereas a_0_ is the invariable constant. The most important issue is what kinds of anthropometric factors should be introduced as explanatory variables deciding SPs. In addition to age (A), height (H) has universally been accepted as the principal explanatory variable predicting SPs. Other anthropometric variables, such as body weight (BW), body mass index (BMI), body fat mass (BFM, defined as multiplying BW by fat percentage of body mass (%FAT)), fat-free mass (FFM), and body surface area (BSA), have also been taken as explanatory variables deciding SPs [6]–[19]. BW is important because it is the sum of the various constituents forming the body, including respiratory muscles that influence a variety of pulmonary function parameters [7]–[9], [21]. BFM may also play a significant role because fat accumulation along central and peripheral airways elicits airway narrowing and leads to a decrease in various SPs [7], [22]. FFM is important because respiratory muscles are contained in FFM. In the present study, however, we did not measure FFM. This is because, for estimating FFM, the skinfold thickness at various regions of the body should be measured [23], the method being time-consuming and not fitted for medical checkups subject to many persons. Instead, we assumed that the difference between BW and BFM might act as the component reflecting FFM in a first approximation. Although some authors used BMI and BSA as explanatory variables for predicting SPs [7]–[9], [10], [11], [18], we considered that the effects of these two variables on SPs would be involved in those of H and BW. This is due to the fact that BMI and BSA are expressed as BW/H^2^ and c_0_⋅(BW)^c1^⋅(H)^c2^ (c_0_, c_1_, and c_2_: constants), respectively. Taking the logarithm of BMI or BSA, each value is converted to the logarithmic additive function as: 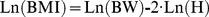
(3)




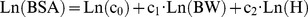
(4)


Eqs. (3) and (4) certainly indicate that the effect of BMI or BSA on SPs is partitioned into the effects of BW and H. Based on these reasons, we adopted age (A), height (H), body weight (BW), and body fat mass (BFM) as explanatory variables for predicting reference means of various spirometric parameters in the present study. Thus, the regression model for a certain SP was assumed the following:
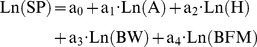
(5)


We decided the coefficients of a_i_ (i = 0 to 4) in each age group of either gender by applying the classical multiple-regression analysis with least-squares minimization. Overall agreement between the predicted reference means and observed values was judged by the coefficient of determination adjusted for degrees of freedom (adjusted-R^2^). Normality for the distribution of residuals of each Ln(SP) was examined using the Kolmogorov-Smirnov and Shapiro-Wilk tests. These tests indicated that the residuals between the logarithm-transformed forms of observed values and those of the predicted reference means did not follow the normal distribution for most of the spirometric parameters. Therefore, we did not calculate the lower limit of normal (LLN) of each Ln(SP) from the standard deviation of residuals (RSD). Instead, we determined it from the 5^th^ percentile of distribution of the residuals. The disparity between the log-transformed value of predicted reference mean and LLN was defined as ΔLLN as follows:
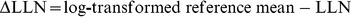
(6)


All calculations were performed using the IBM SPSS Statistics (Version 21.0, SPSS Inc., an IBM Co., NY, USA). Unless otherwise specified, the values were expressed as the means ± standard deviations. A p-value lower than 0.05 was deemed to be statistically significant.

## Results

In the male participants, H was the maximum in young-age group, whereas BMI displayed the largest value in the middle-age group (Table 1). The values of BW, %FAT, and BFM in young-age and middle-age groups were higher than those in the advanced-age group. In the men, all spirometric parameters were significantly different between three groups, and the highest value was observed in the young-age group (Table 1).

Somewhat different from the male participants, H was decreased and BMI increased with aging in the female participants (Table 2). BW or BFM reached the maximum level in the middle-age group, whereas %FAT displayed the highest value in the middle-age and advanced-age groups. In the female participants, spirometric parameters other than PEF revealed trends identical to those investigated in the male participants (Table 2).

Among the partial regression coefficients of a_0_, a_1_, a_2_, a_3_, and a_4_ determined in the young-age, middle-age, and advanced-age groups of both genders, only the values reaching statistical significance are depicted in Tables 3, 4, and 5, respectively.

**Table 3:** Partial-regression coefficients of log-transformed equations predicting the reference means of spirometric parameters in the young-age male and female groups.

	Constant	Ln(Age)	Ln(H)	Ln(BW)	Ln(BFM)	R^2^	ΔLLN
Ln(FVC)							
M	−7.093		1.656			0.270	0.408
F	−4.434		0.980	0.153		0.264	0.264
Ln(FEV_1_)							
M	−6.746		1.551			0.240	0.439
F	−4.292	−0.117	1.059	0.082		0.250	0.271
Ln(FEV_1_/FVC)							
M	4.926	−0.080		−0.054		0.150	0.117
F	4.891	−0.107			−0.030	0.199	0.135
Ln(PEF)							
M	−2.373		0.871			0.125	0.456
F	0.797			0.244		0.154	0.373
Ln(FEF_50_)							
M	−2.231	−0.251	0.889			0.114	0.552
F	−1.244	−0.276	0.603	0.105		0.139	0.457
Ln(FEF_75_)							
M	−5.671	−0.531	1.623		−0.153	0.190	0.892
F	−5.051	−0.665	1.579		−0.142	0.223	0.775

Ln: natural logarithm. M: male. F: female. H: height. BW: body weight. BFM: body fat mass. R^2^: coefficient of determination adjusted for degrees of freedom. ΔLLN  =  reference mean – LLN in Ln form. Only the coefficients with statistical significance are presented (at least p<0.04).

**Table 4:** Partial-regression coefficients of log-transformed equations predicting the reference means of spirometric parameters in the middle-age male and female groups.

	Constant	Ln(Age)	Ln(H)	Ln(BW)	Ln(BFM)	R^2^	ΔLLN
Ln(FVC)							
M	−5.921	−0.178	1.580		−0.051	0.318	0.378
F	−5.120	−0.147	1.348			0.276	0.268
Ln(FEV_1_)							
M	−4.625	−0.263	1.265	0.138	−0.108	0.318	0.385
F	−4.152	−0.245	1.191			0.291	0.272
Ln(FEV_1_/FVC)							
M	5.433	−0.079	−0.140		−0.008	0.142	0.118
F	5.793	−0.099	−0.233	0.063	−0.030	0.166	0.119
Ln(PEF)							
M	−1.990	−0.136	0.895			0.155	0.485
F	0.749	−0.079		0.415	−0.119	0.122	0.384
Ln(FEF_50_)							
M	−1.381	−0.326	0.776			0.160	0.557
F	1.216	−0.316		0.347	−0.081	0.155	0.520
Ln(FEF_75_)							
M	−1.493	−0.774	0.764	0.329	−0.260	0.260	0.731
F	1.993	−0.918		0.560	−0.280	0.276	0.681

Symbols are as depicted in Table 3. The coefficients presented are statistically significant (at least p<0.03).

**Table 5:** Partial-regression coefficients of log-transformed equations predicting the reference means of spirometric parameters in the advanced-age male and female groups.

	Constant	Ln(Age)	Ln(H)	Ln(BW)	Ln(BFM)	R^2^	ΔLLN
Ln(FVC)							
M	−3.976	−0.420	1.387		0.034	0.345	0.321
F	−3.970	−0.640	1.523			0.353	0.288
Ln(FEV_1_)							
M	−3.201	−0.579	1.318		−0.034	0.372	0.317
F	−2.498	−0.804	1.320			0.361	0.309
Ln(FEV_1_/FVC)							
M	4.989	−0.150				0.171	0.114
F	6.207	−0.164	−0.255	0.033		0.191	0.113
Ln(PEF)							
M	−2.007	−0.245	0.985			0.186	0.450
F	−1.182	−0.536	0.991		0.053	0.238	0.409
Ln(FEF_50_)							
M	0.697	−0.976	0.898			0.267	0.569
F	5.496	−1.281		0.217		0.289	0.604
Ln(FEF_75_)							
M	−1.115	−1.530	1.659	−0.274		0.314	0.717
F	3.016	−1.705	0.737			0.289	0.723

Symbols are the same as shown in Table 3. All coefficients presented are statistically significant (at least p<0.02).

### Relationship between the anthropometric variables and spirometric parameters of the young-age group (Table 3)

Age did not act as a significant determinant on FVC and PEF in both genders as well as on FEV_1_ in male participants. However, age was a decisive factor for FEV_1_ in female participants and FEV_1_/FVC as well as flow parameters, including FEF_50_ and FEF_75_, in both genders.

H had a substantial impact on FVC, FEV_1_, and the flow parameters of FEF_50_ and FEF_75_ in both genders. However, it did not have a significant impact on FEV_1_/FVC in both genders and PEF in female participants.

BW functioned as one of the determinants on FEV_1_/FVC in male participants and FVC, FEV_1_ PEF, and FEF_50_ in female participants.

BFM had an appreciable impact on FEV_1_/FVC in female participants and FEF_75_ in both genders. However, there was no spirometric parameter simultaneously influenced by BW and BFM.

### Middle-age group (Table 4)

Unlike the young-age group, age played an important role in deciding all spirometric parameters in the middle-age group irrespective of gender.

Although H acted as a significant explanatory variable determining all spirometric parameters in male participants, it had no significant influence on the flow parameters of PEF, FEF_50_, and FEF_75_ in female participants.

BW and BFM concurrently exerted a considerable influence on FEV_1_ and FEF_75_ in male participants as well as FEV_1_/FVC and all flow parameters in female participants. The direction of contributions of BW and BFM was opposite, i.e., BW increased, whereas BFM decreased, a given spirometric parameter. However, apart from BW, BFM appreciably contributed to determination of the FVC and FEV_1_/FVC of male participants.

### Advanced-age group (Table 5)

Similar to the middle-age group, age played a significant role for all spirometric parameters independent of gender in the advanced-age group. It should be noted that the decline of pulmonary function with age was consistently larger in the advanced-age group than in other age groups regardless of the spirometric parameter or the gender.

H functioned as a decisive factor for many spirometric parameters in advanced-age group, but it had no significant influence on FEV_1_/FVC in male participants and FEF_50_ in female participants.

In contrast to the middle-age group, the concurrent contribution of BW and BFM to the spirometric parameters was not investigated in the advanced-age group. BW decreased FEF_75_ in male participants but increased FEV_1_/FVC and FEF_50_ in female participants. BFM increased FVC in male participants as well as PEF in female participants but decreased FEV_1_ in male participants.

### Age- and gender-dependent contributions of anthropometric variables to spirometric parameters (Figs. 1 to 9)

**Figure 1:**
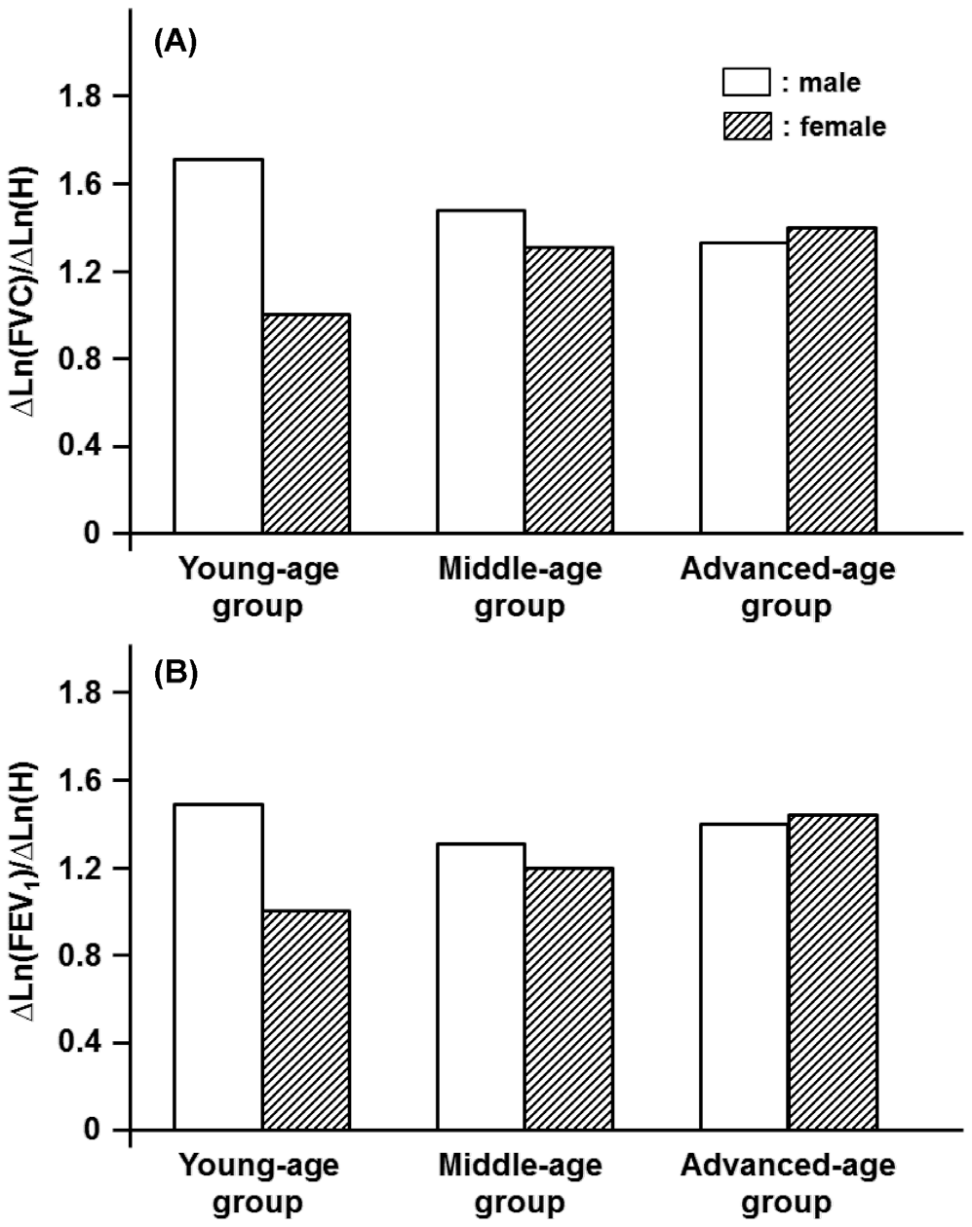
Age-specific effects of height (H) on decision of FVC and FEV_1_ estimated for three different age groups of either males or females. (A): Partial regression coefficients of Ln(H) for reference means of Ln(FVC) in young-, middle-, and advanced-age groups of both genders. Partial regression coefficients of Ln(H) for Ln(FVC) are denoted as ΔLn(FVC)/ΔLn(H). (B): Partial regression coefficients of Ln(H) for Ln(FEV_1_) are designated as ΔLn(FEV_1_)/ΔLn(H).

**Figure 2:**
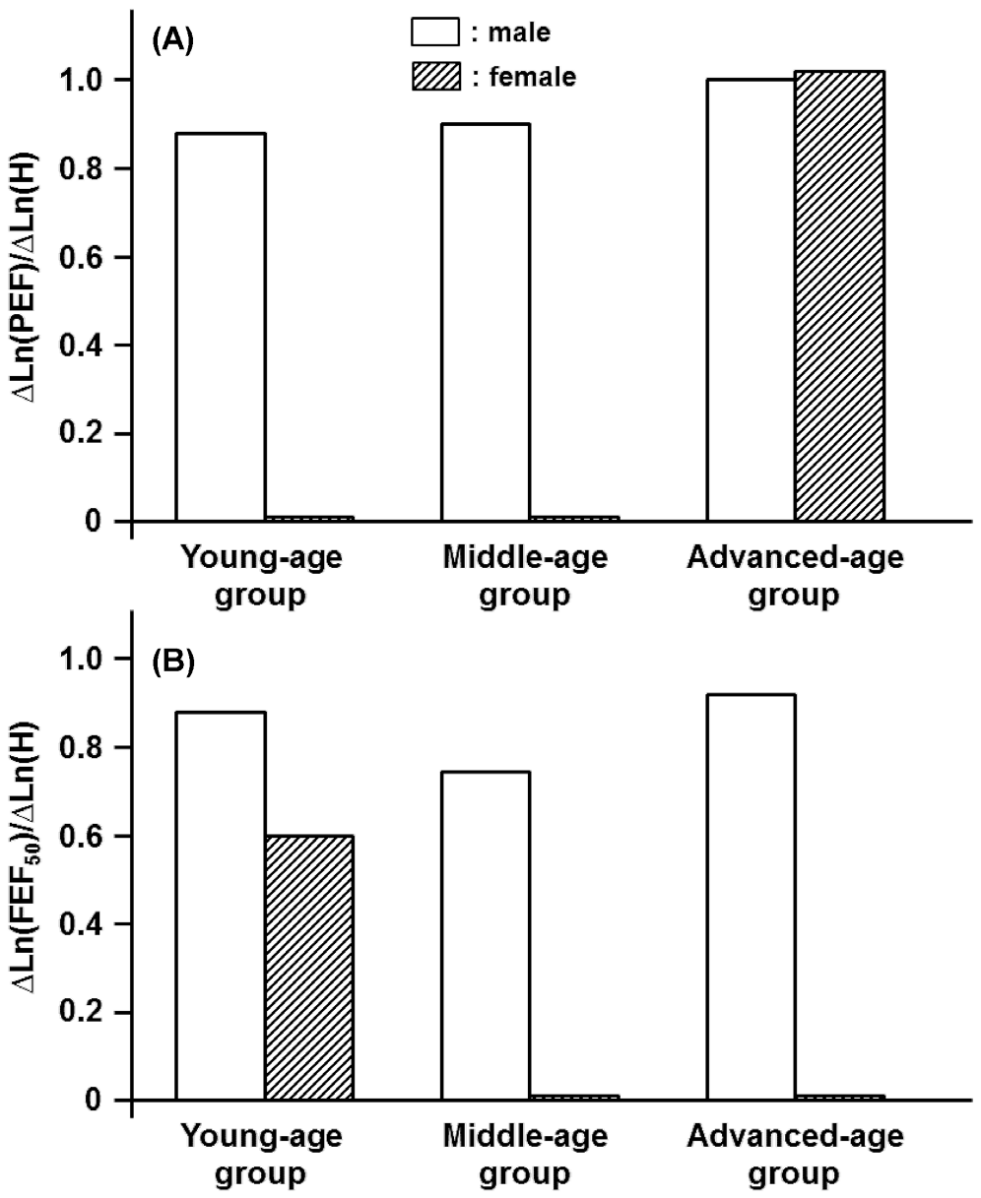
Age-specific impacts of height (H) on PEF and FEF_50_ estimated for three different age groups of both men and women. (A): Partial regression coefficients of Ln(H) for reference means of Ln(PEF) in young-, middle-, and advanced-age groups of either gender. Partial regression coefficients of Ln(H) for ΔLn(PEF) are defined as ΔLn(PEF)/ΔLn(H). (B): Partial regression coefficients of Ln(H) for Ln(FEF_50_) are defined as ΔLn(FEF_50_)/ΔLn(H).

**Figure 3:**
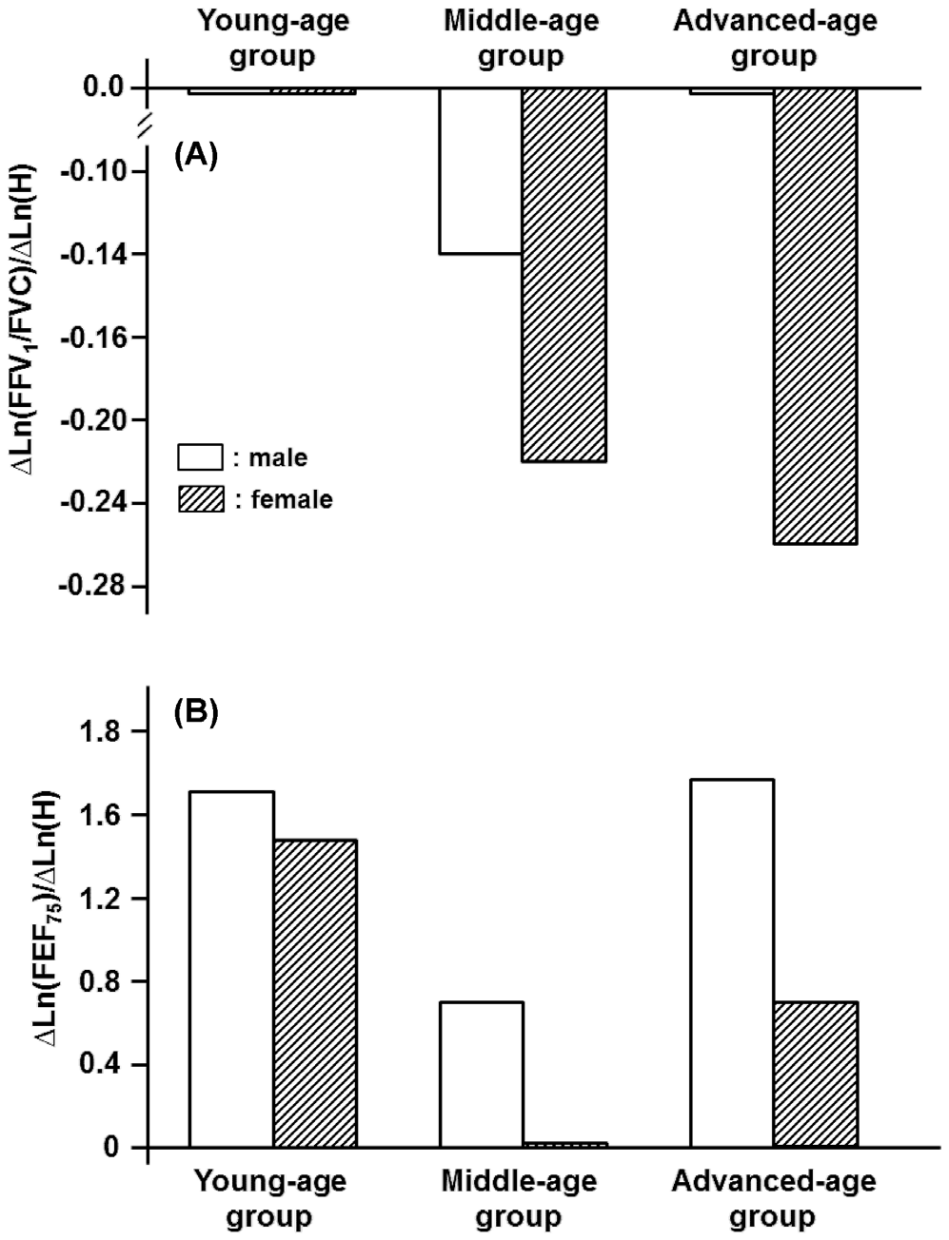
Age-specific effects of height (H) on decision of FEV_1_/FVC and FEF_75_ estimated for three different age groups of male and female participants. (A): Partial regression coefficients of Ln(H) for reference means of Ln(FEV_1_/FVC) in young-, middle-, and advanced-age groups of either gender. Partial regression coefficients of Ln(H) for Ln(FEV_1_/FVC) are denoted as ΔLn(FEV_1_/FVC)/ΔLn(H). (B): Partial regression coefficients of Ln(H) for Ln(FEF_75_) are designated as ΔLn(FEV_75_)/ΔLn(H).

Depending on gender and age, the anthropometric variables of H, BW, and BFM were inextricably linked with spirometric parameters in a qualitatively and/or quantitatively different manner. One exception was the effect of H on FVC and FEV_1_. For these parameters, the impact of H was relatively homogeneous and approximately independent of age in both genders (Fig. 1). The identical tendency, i.e., the constant partial regression coefficient of H with respect to age, approximately held for PEF and FEF_50_ but not for FEV_1_/FVC and FEF_75_ for the male participants (Figs. 2, 3). On the other hand, the partial regression coefficients of H for spirometric parameters, except for FVC and FEV_1_, were not constant and changed significantly with age in the female participants (Figs. 1-3).

**Figure 4:**
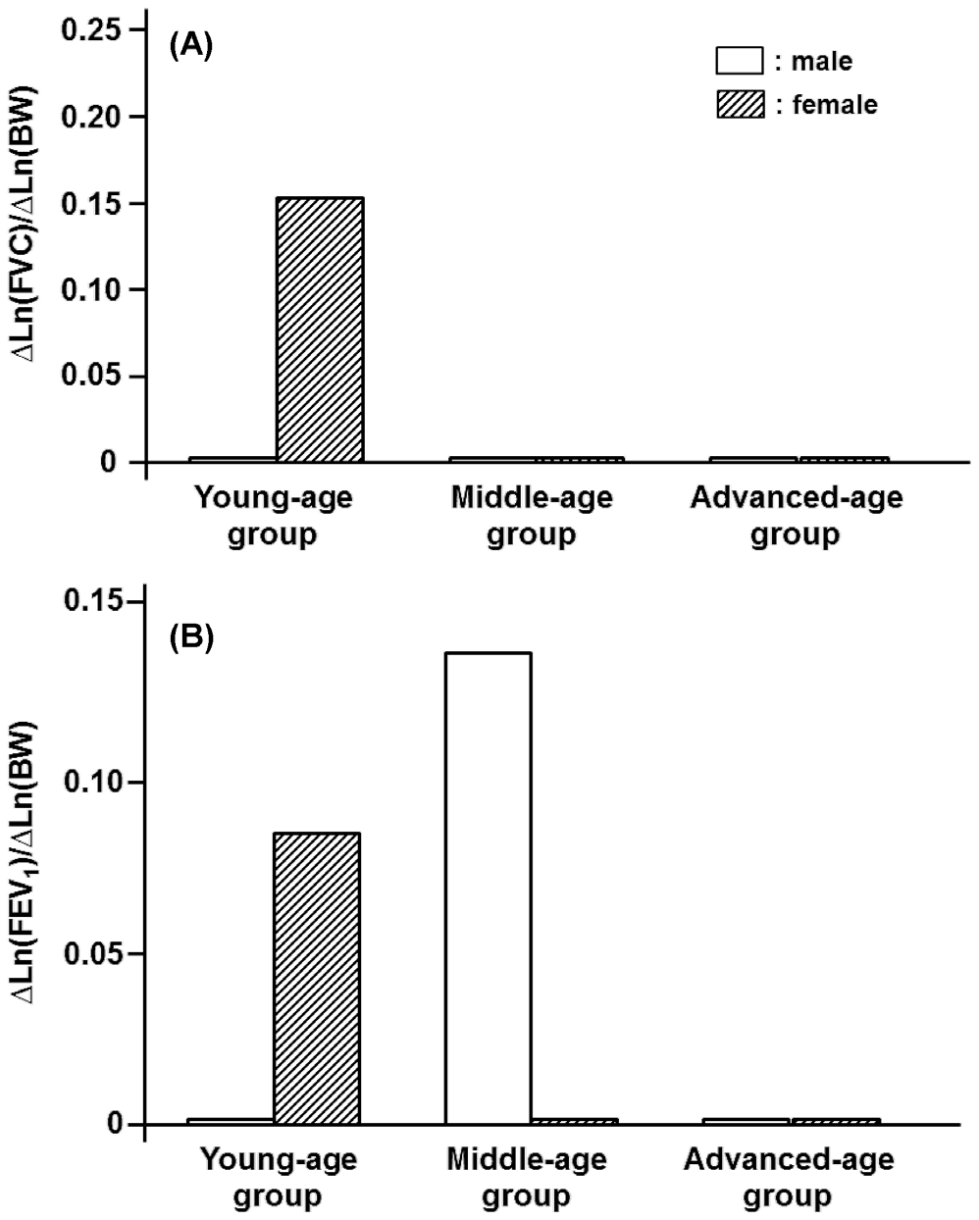
Age-specific contributions of body weight (BW) to FVC and FEV_1_ estimated for three different age groups of both genders. (A): Partial regression coefficients of Ln(BW) for reference means of Ln(FVC) in young-, middle-, and advanced-age groups of either gender. They are designated as ΔLn(FVC)/ΔLn(BW). (B): Partial regression coefficients of Ln(BW) for Ln(FEV_1_) are designated as ΔLn(FEV_1_)/ΔLn(BW).

**Figure 5:**
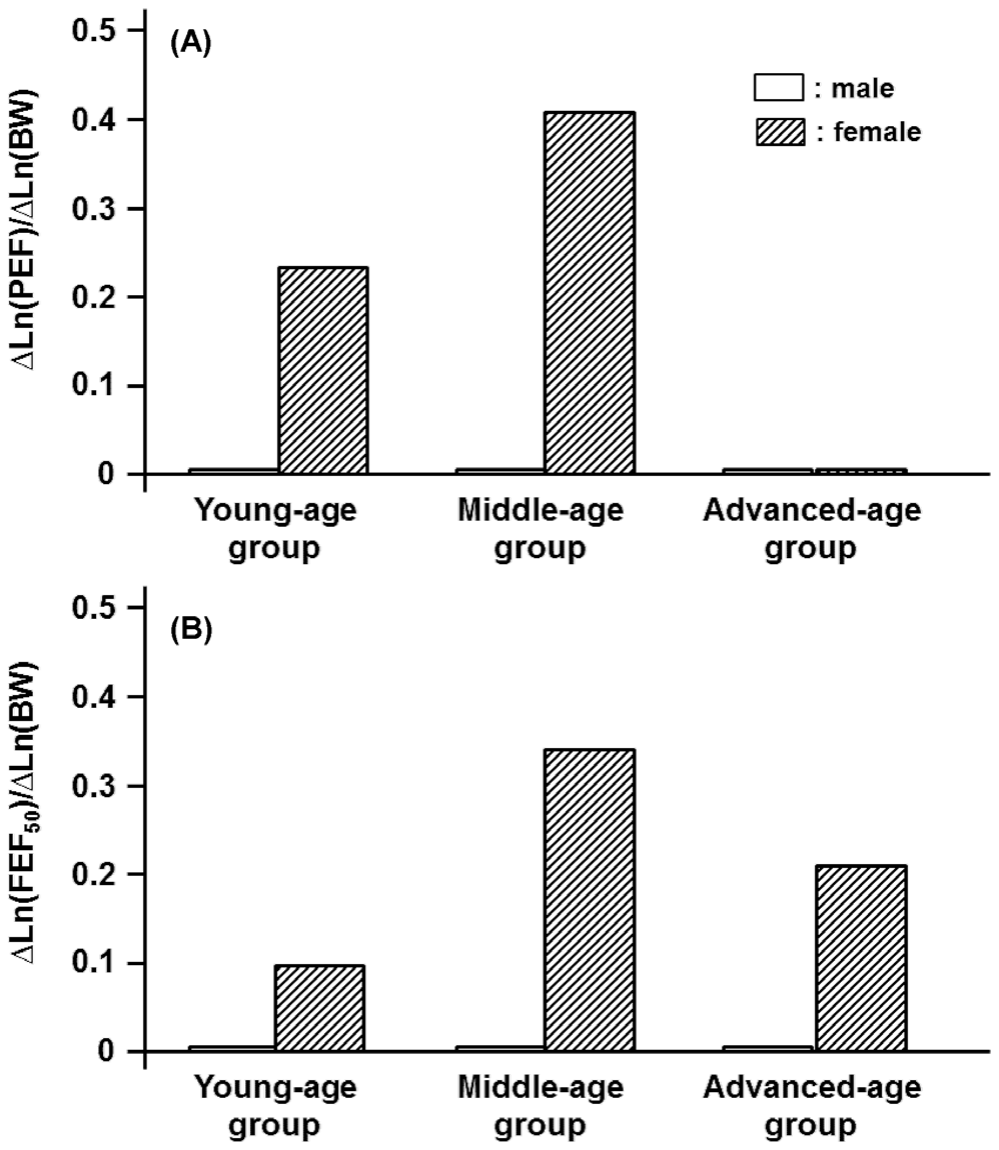
Age-specific impacts of body weight (BW) on PEF and FEF_50_ estimated for three different age groups of male and female participants. (A): Partial regression coefficients of Ln(BW) for the reference means of Ln(PEF) in young-, middle-, and advanced-age groups of both genders. Partial regression coefficients of Ln(BW) for Ln(PEF) are defined as ΔLn(PEF)/ΔLn(BW). (B): Partial regression coefficients of Ln(BW) for Ln(FEF_50_) are defined as ΔLn(FEF_50_)/ΔLn(BW).

**Figure 6:**
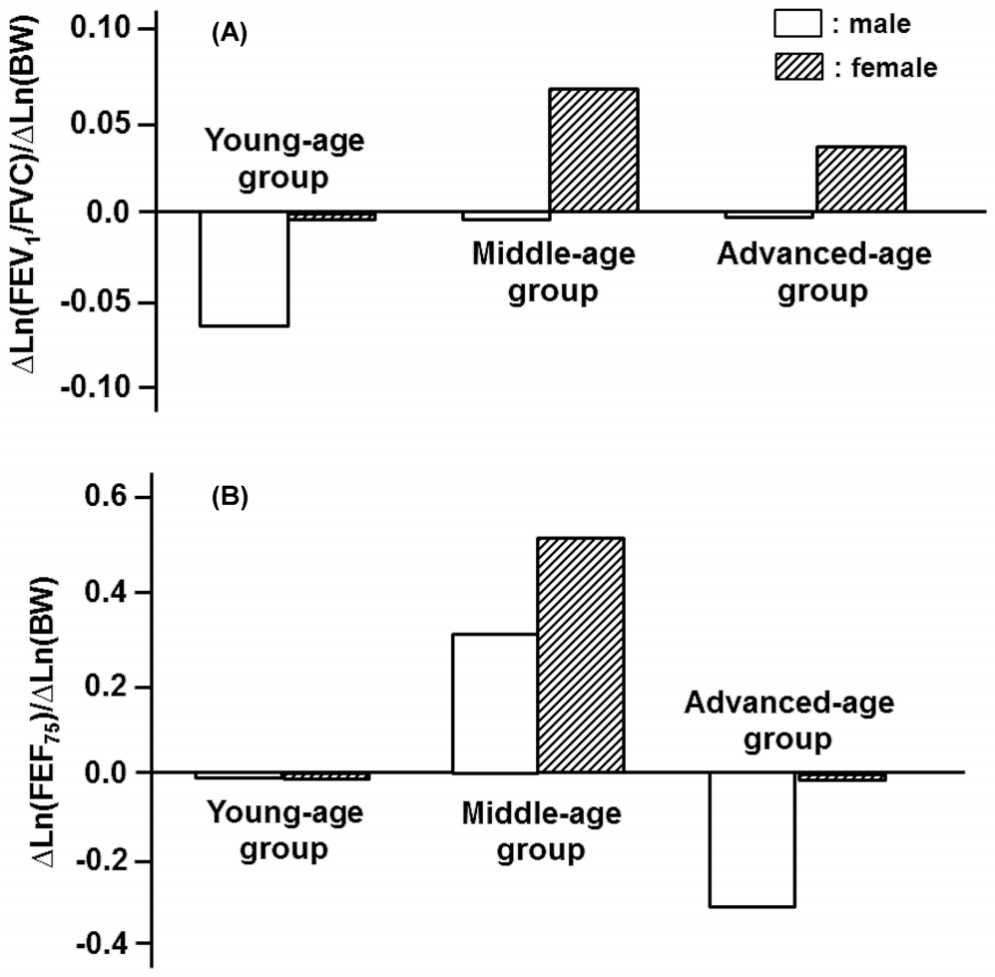
Age-specific contributions of body weight (BW) to decision of FEV_1_/FVC and FEF_75_ estimated for three different age groups of both genders. (A): Partial regression coefficients of Ln(BW) for reference means of Ln(FEV_1_/FVC) in young-, middle-, and advanced-age groups of both genders. They are designated as ΔLn(FEV_1_/FVC)/ΔLn(BW). (B): Partial regression coefficients of Ln(BW) for Ln(FEF_75_) are designated as ΔLn(FEF_75_)/ΔLn(BW).

BW exerted little influence on FVC, PEF, and FEF_50_ in any of the age groups in the male participants (Figs. 4, 5). For the male participants, however, BW had appreciable impacts on FEV_1_, FEV_1_/FVC and FEF_75_ in some age groups (Figs. 4, 6). In the female participants, BW played an important role in determining FVC or FEV_1_ only in the young-age group (Fig. 4). The FEV_1_/FVC and the flow parameters of PEF, FEF_50_, and FEF_75_ in the female groups were substantially influenced by BW in an age-dependent manner, with the maximum effect being detected in the middle-age group (Figs. 5, 6).

**Figure 7:**
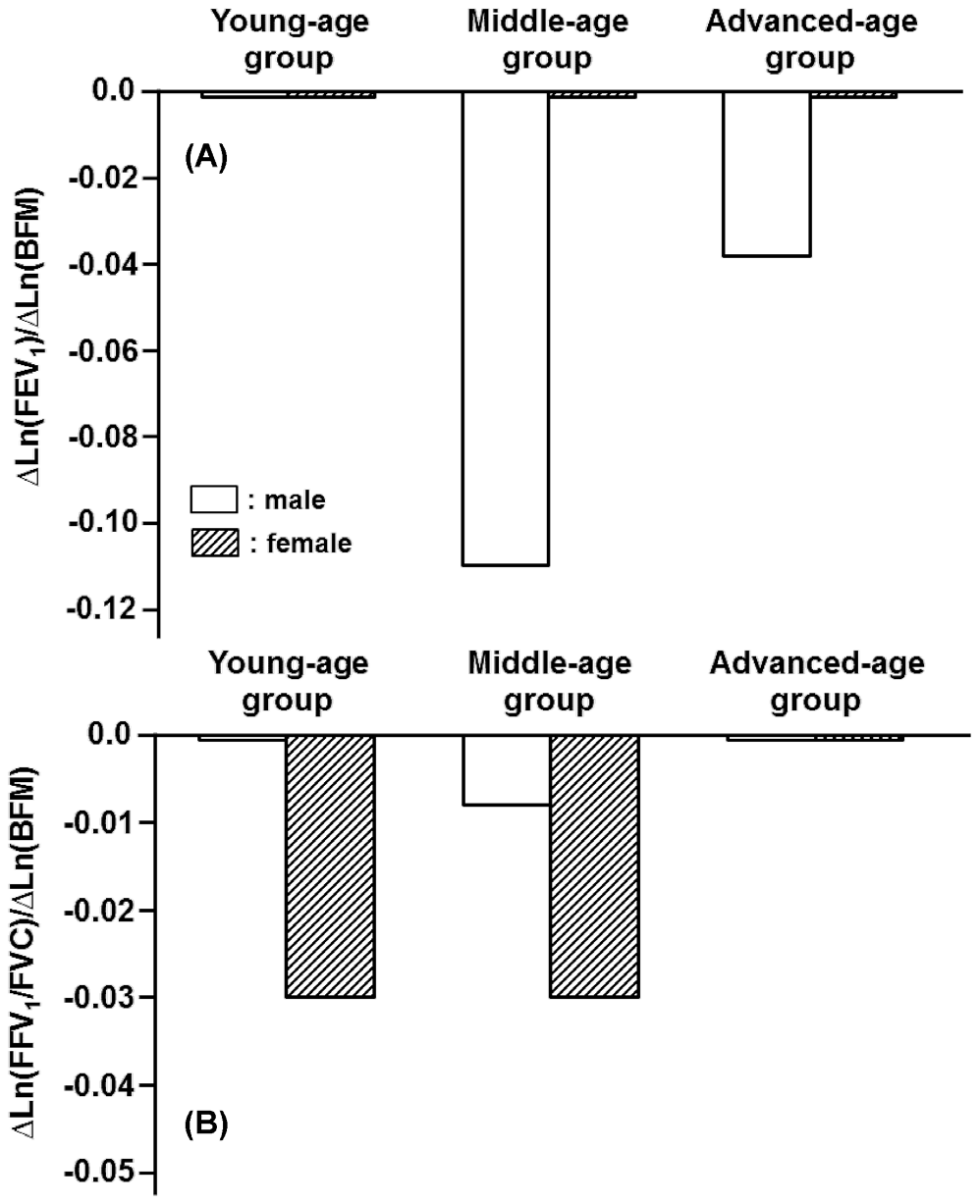
Age-specific impacts of body fat mass (BFM) on FEV_1_ and FEV_1_/FVC estimated for three different age groups of both men and women. (A): Partial regression coefficients of Ln(BFM) for reference means of Ln(FEV_1_) in young-, middle-, and advanced-age groups of either gender, which were expressed as ΔLn(FEV_1_)/ΔLn(BFM). (B): Partial regression coefficients of Ln(BFM) for Ln(FEV_1_/FVC), being defined as ΔLn(FEV_1_/FVC)/ΔLn(BFM).

**Figure 8:**
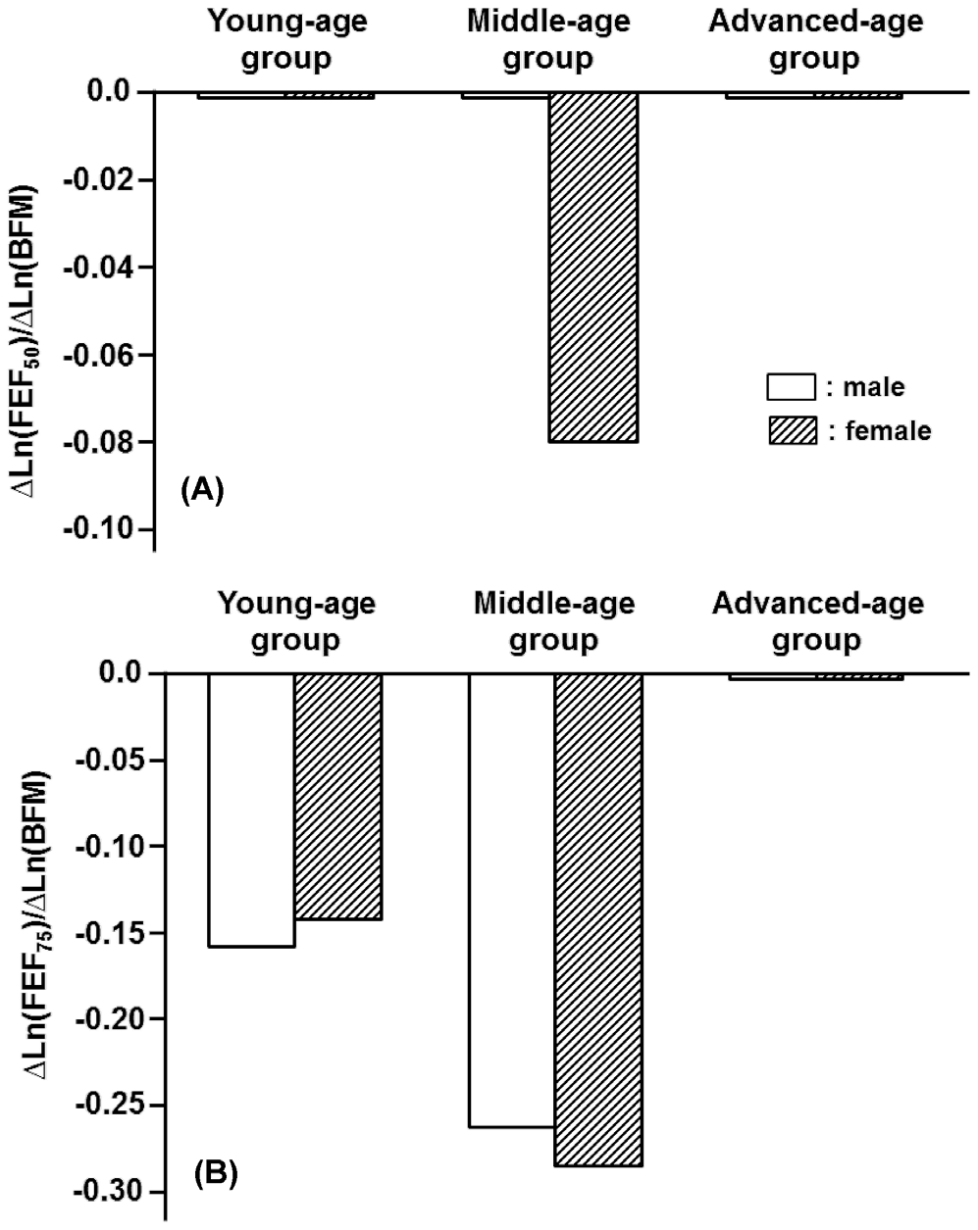
Age-specific impacts of body fat mass (BFM) on deciding FEF_50_ and FEF_75_ estimated for three different age groups of male and female participants. (A): Partial regression coefficients of Ln(BFM) for reference means of Ln(FEF_50_) in young-, middle-, and advanced-age groups of either gender. They are expressed as ΔLn(FEF_50_)/ΔLn(BFM). (B): Partial regression coefficients of Ln(BFM) for Ln(FEF_75_) are defined as ΔLn(FEF_75_)/ΔLn(BFM).

**Figure 9:**
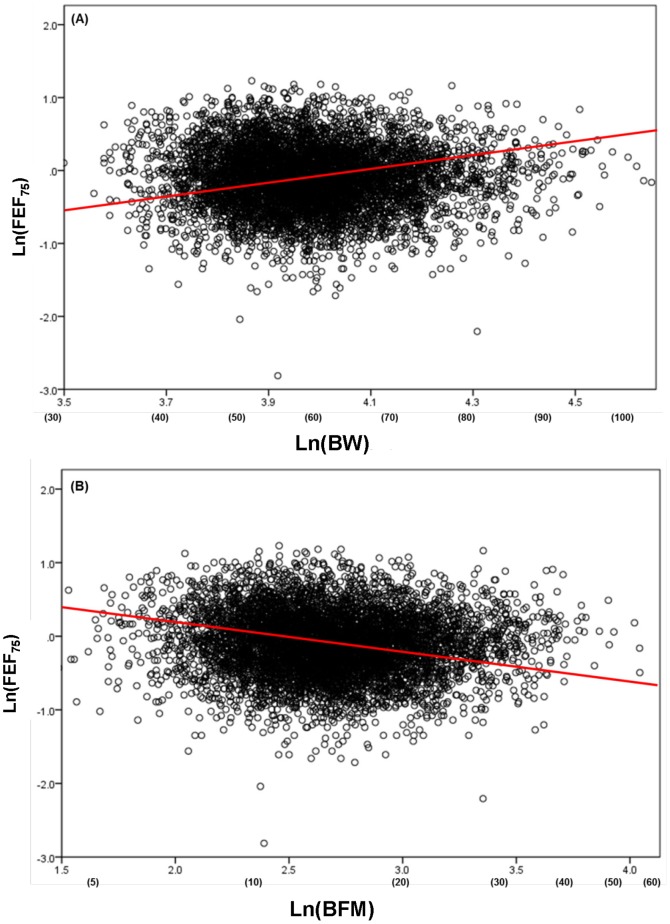
Opposite effects of body weight (BW) and body fat mass (BFM) on FEF_75_ in female middle-age group. (A): Ln(FEF_75_) vs. Ln(BW). Parentheses: absolute values of BW without log-transformation. Red line: slope of the equation predicting reference means of Ln(FEF_75_) from Ln(BW), which is equal to partial regression coefficient shown in Table 4 and Fig. 6-(B). (B): Ln(FEF_75_) vs. Ln(BFM). Parentheses: absolute values of BFM with no log-transformation. Red line: slope of the equation predicting reference means of Ln(FEF_75_) from Ln(BFM), which is equal to partial regression coefficient given in Table 4 and Fig. 8-(B).

Although H and BW generally were factors that increased spirometric parameters, BFM exerted negative impacts on most of the spirometric parameters in an age-dependent fashion (Figs. 7, 8). The concurrent impacts of BW and BFM were specifically detected in the female middle-age group, in which BW functioned as an increasing factor, whereas BFM was as a decreasing factor for a variety of flow parameters (Fig. 9). The influence of BFM on spirometric parameters was weaker in the young- and advanced-age groups irrespective of gender (Fig. 7, 8).

## Discussion

### Critique of the method

Although we applied the multiplicative model similar to that proposed by the GLI [3]–[5], we did not determine the partial regression coefficients for Ln(SP) in terms of the LMS method with smoothing such as penalized beta-spline [24], [25], i.e., more generalized additive, linear model describing location, scale, and shape (GAMLSS) of a given dataset. Instead, we determined the coefficients with the method of classical multiple-regression analysis of least-squares minimization without smoothing. The reason for this simplification is that the main purpose of the present study is not to establish the generalized regression equations for SPs applicable for all ages but to explore the age- and gender-associated impacts of each anthropometric factor on each SP.

We should note that it has not been verified whether the age-related importance of anthropometric variables for determining spirometric parameters investigated in the present study holds true for all races and ethnicities because our study population consisted entirely of nonsmoking healthy Japanese adults.

### Aging- and gender-dependent contributions of age to spirometric parameters

Although age was not a determinant on FVC and PEF in the young-age groups of both genders and FEV_1_ in the male young-age group, age played an important role in deciding other spirometric parameters in any age groups irrespective of the gender. It should be noted that the decline of pulmonary function with age was consistently larger in the advanced-age group than in other age groups regardless of the spirometric parameter or the gender, indicating that the degree of effects of age on spirometric parameters would differ depending on aging. These facts suggest that the assumption of aging-independent, constant partial regression coefficients of age are not acceptable for any spirometric parameters in both genders.

### Age- and gender-dependent contributions of anthropometric variables to spirometric parameters

We demonstrated that the anthropometric variables of H, BW, and BFM were linked with spirometric parameters generally in an age-dependent manner. However, the impact of H on FVC and FEV_1_ was relatively homogeneous and approximately independent of age in both genders, indicating that the assumption of an age-independent, constant value of the partial regression coefficient of H is only acceptable for the equation predicting the reference mean of FVC or FEV_1_ irrespective of the gender. The identical assumption, i.e., the constant partial regression coefficient of H with regard to age, approximately held for PEF and FEF_50_ for the male participants. On the other hand, the partial regression coefficients of H for most of the spirometric parameters in the female participants were changed significantly with age, suggesting that the age-dependent contribution of H to the spirometric parameters is more evident in the female participants than in the male participants.

For the male participants, BW exerted little influence on FVC, PEF, and FEF_50_ in any of the age groups, allowing us to set the partial regression coefficients of BW for these spirometric parameters to zero, i.e., the homogeneous effects of BW on FVC, PEF, and FEF_50_ in the male participants. For the male participants, however, BW had appreciable impacts on FEV_1_, FEV_1_/FVC and FEF_75_ in some age groups, indicating that the assumption of the age-independent, constant partial regression coefficients of BW was not acceptable for these spirometric parameters. For the female participants, BW played a substantial role in determining all of the spirometric parameters in an age-dependent manner. Joining the findings observed for male and female participants, we considered that, although the impact of BW on spirometric parameters were very inhomogeneous and changed distinctly depending on gender and age, they were more conspicuous for the female participants than for the male participants.

In contrast with H and BW, BFM exerted negative influences on most of the spirometric parameters in an age-dependent fashion. In the female middle-age group, BW functioned as an increasing factor, whereas BFM was as a decreasing factor for a variety of flow parameters, indicating that the negative effect of BFM on certain spirometric parameters could be offset by the positive effect of BW. The importance of BW and BFM in the female middle-age group is supported by the fact that physique changes with aging, leading to both BW and BFM reaching the maximums in this female age group (Table 2). On the other hand, the influence of BW and BFM on spirometric parameters appeared to be weak in the advanced-age population irrespective of gender. This is also explained by the finding that BW and BFM generally decreased with aging, reaching the minimums or the lower levels in the advanced-age population (Tables 1, 2).

### Importance of body weight and body fat mass in determining spirometric parameters

BW is the sum of the various constituents, including respiratory muscles and fat deposition in the thoracic cavity and around the airway. Respiratory muscles influence the maximal respiratory pressures, and, hence, a variety of pulmonary function parameters such as inspiratory capacity, FVC, FEV_1_, and PEF [7]–[9], [21]. Fat components represented by BFM influence the lung volumes, the work of breathing, and, in some circumstances, the airway caliber [7], [22]. Fat accumulation along a large airway may cause central airway narrowing, leading to a decrease in FVC, FEV_1_, and/or PEF. On the other hand, fat deposition along a small airway may elicit peripheral airway narrowing, leading to reduction in flow parameters such as FEF_50_ and FEF_75_. We found that the negative impact of BFM on the spirometric parameter was age-dependent and evident for FVC (male), FEV_1_ (male), FEV_1_/FVC (both genders), PEF (female), FEF_50_ (female), or FEF_75_ (both genders) in the middle-age group. Our findings are partially consistent with those reported by Cotes et al. [9], who demonstrated that the fat percentage of body mass (%FAT) plays a negative role in determining FVC and FEV_1_ in both genders. Unfortunately, however, they did not address the age-dependent contribution of %FAT to the spirometric parameters. Interestingly, we found that, in contrast to BFM, BW had a positive impact on most of the spirometric parameters, though BFM was one of the components forming BW. This peculiar finding may be explained, in part, by the fact that the content of respiratory muscles is among the components of BW as well. Our findings are in accordance with the study of Pistelli et al. [18], who demonstrated the positive effect of BMI on many spirometric parameters, but our findings are seemingly inconsistent with that of Chen et al. [6] and Chinn et al. [7], both of whom demonstrated a weight-gain-associated decline in lung function. A longitudinal study done by Chen et al. [6] showed that each kg of weight-gain was associated with an excess loss of 26 mL in FVC and 23 mL in FEV_1_ in men, and 14 mL and 9 mL respectively in women. Chinn et al. [7] revealed that FVC and FEV_1_ were decreased by about 20 mL per kg-gain of BW in a longitudinal study for male shipyard workers. As pointed out by Chinn et al. [7], however, the increase in BW of a certain person in a longitudinal observation is generally caused by the increase in BFM but not by the increase in FFM. In other word, the effect of weight-gain on lung function in a longitudinal observation reflects the effect of increased BFM at a relatively constant level of FFM. Therefore, we considered that the findings reported by Chen et al. [6] and Chinn et al. [7] were not inconsistent with our findings, in which BFM was found to have a negative impact on lung function.

### Clinical implication of the present study

We found that, in addition to age and height, body weight and body fat mass had substantial impacts on deciding the reference means of various spirometric parameters. Furthermore, we demonstrated that the effects of all explanatory variables introduced in the present study were conspicuously age-dependent. Therefore, we considered that our findings would serve as a warning to the reference equations reported over several decades, in which aging-independent contributions of two explanatory variables, including age and height, to spirometric parameters were simply assumed. Since spirometric parameters are measured under a forced expiratory maneuver, the effect of the variables involving the respiratory muscles should be taken into account. For this purpose, fat-free mass (FFM) or bone-free lean body mass (BF-LBM) deserves a good indicator [17]. It is not easy, however, to measure these variables in a cohort consisting of numerous subjects. Therefore, we used body weight as a substitute for them, in which respiratory muscles are contained. However, we should notice that body weight has a disadvantage, i.e., it includes body fat mass (BFM) as well. In an obese subject, the excessive body weight caused by increased BFM will conceal the positive effect of respiratory muscles in association with disclosing the negative effect of fat deposition in the thoracic and abdominal cavities, which leads to narrowing the airway calibers and inhibiting the diaphragm movement. We consider that, to defeat the disadvantageous effect of body weight caused by increased BFM, it is indispensable to concurrently estimate the independent effects of body weight and that of BFM, like the present study did.

Although we did not estimate the quantitative difference between the predicted means of spirometric parameters calculated from our equations (aging-dependent differences in partial regression coefficients of age, height, body weight, and body fat mass) and those calculated under a simple assumption of constant partial regression coefficients of age and height with regard to aging, we believe that the latter may exert an erroneous impact on the predicted means of spirometric parameters.

## Conclusions

This is the first large clinical trial demonstrating the important roles of age-dependent contributions of anthropometric variables, including height, body weight, and body fat mass, to various spirometric parameters. Furthermore, we demonstrated that the age-related effects of anthropometric variables on spirometric parameters were gender-dependent, and these effects were more evident in the female participants than in the male participants. The findings in the present study suggest that the assumption of constant partial regression coefficients for anthropometric variables with respect to age may result in a substantial error when predicting the reference means of various spirometric parameters, especially in women.

## References

[1] Lung Function in Growth and Aging (2012) Publish reference values. Available: www.lungfunction.org/publishedreferencevalues.html.

[2] Stanojevic S, Wade A, Stocks J, Hankinson J, Coates AL, et al (2008) Reference ranges for spirometry across all ages. Am J Respir Crit Care Med 177: 253–260. 18006882 10.1164/rccm.200708-1248OC PMC2643211

[3] Quanjer PH, Stanojevic S, Stocks J, Cole TJ (2012) GAMLSS in action - annotated examples of working with R and GAMLSS. Available: www.spirxpert.com/download/GAMLSS-in-action.zip.

[4] Quanjer PH, Stanojevic S, Cole TJ (2012) Multi-ethnic reference values for spirometry for the 3–95 year age range: the global lung function equations. Eur Repir J doi:10.1183/09031936.00080312. 10.1183/09031936.00080312 PMC3786581 22743675

[5] Stocks J, Baur X, Hall G, Culver B, GLI ERS Task Force (TF-2009-03 to establish improved Lung Function Reference Values) (2012) Implementing GLI 2012 lung function regression equations. Available: www.lungfunction.org/files/pdf.

[6] Chen Y, Horne SL, Dosman JA (1993) Body weight and weight gain related to pulmonary function decline in adults: a six year follow up study. Thorax 48: 375–380. 8511735 10.1136/thx.48.4.375 PMC464436

[7] Chinn DJ, Cotes JE, Reed JW (1996) Longitudinal effects of change in body mass on measurements of ventilatory capacity. Thorax 51: 699–704. 8882076 10.1136/thx.51.7.699 PMC472492

[8] Cotes JE, Dabbs JM, Hall AM, Heywood HC, Laurence KM (1979) Sitting height, fat-free mass and body fat as reference variables for lung function in healthy British children: comparison with stature. Ann Hum Biol 6: 307–314. 575278 10.1080/03014467900003691

[9] Cotes JE, Chin DJ, Reed JW (2001) Body mass, fat percentage, and fat free mass as reference variables for lung function: effects on terms for age and sex. Thorax 56: 839–844. 11641507 10.1136/thorax.56.11.839 PMC1745971

[10] García-Río F, Pino JM, Dorgham A, Alonso A, Villamor J (2004) Spirometric reference equations for European females and males aged 65–85 yrs. Eur Respir J 24: 397–405. 15358698 10.1183/09031936.04.00088403

[11] García-Río F, Dorgham A, Pino JM, Villasante C, Garcia-Quero C, et al (2009) Lung volume reference values for women and men 65 to 85 Years of Age. Am J Respir Crit Care Med 180: 1083–1091. 19745204 10.1164/rccm.200901-0127OC

[12] Gore CJ, Crockett AJ, Pederson DG, Booth ML, Bauman A, et al (1995) Spirometric standards for healthy adult lifetime nonsmokers in Australia. Eur Respir J 8: 773–782. 7656950

[13] Hall AM, Heywood C, Cotes JE (1979) Lung function in healthy British women. Thorax 34: 359–365. 483211 10.1136/thx.34.3.359 PMC471075

[14] Hankinson JL, Odencrantz JR, Fedan KB (1999) Spirometric reference values from a sample of the general U.S. population. Am J Respir Crit Care Med 159: 179–187. 9872837 10.1164/ajrccm.159.1.9712108

[15] Jenkins SC, Moxham J (1991) The effect of mild obesity on lung function. Respir Med 85: 309–311. 1947368 10.1016/s0954-6111(06)80102-2

[16] Mengesha YA, Mekonnen Y (1985) Spirometric lung function tests in normal non-smoking Ethiopian men and women. Thorax 40: 465–468. 4024008 10.1136/thx.40.6.465 PMC460106

[17] Mohamed EI, Maiolo C, Iacopino L, Pepe M, Di Daniele N, et al (2002) The impact of body-weight components on forced spirometry in healthy Italians. Lung 180: 149–159. 12177729 10.1007/s004080000089

[18] Pistelli F, Bottai M, Viegi G (2000) Smooth reference equations for slow vital capacity and flow-volume curve indexes. Am J Respir Crit Care Med 161: 899–905. 10712340 10.1164/ajrccm.161.3.9906006

[19] Wu Y, Zhang Z, Gang B, Love EJ (2009) Predictive equations for lung function based on a large occupational population in North China. J Occup Health 51: 471–477. 19779280 10.1539/joh.l9006

[20] Miller MR, Hankinson J, Brusasco V (2005) Standardisation of spirometry (Series “ATS/ERS Task Force: Standardisation of Lung Function Testing). Eur Respir J 26: 319–338. 16055882 10.1183/09031936.05.00034805

[21] Schoenberg JB, Beck GJ, Bouhoys A (1978) Growth and decay of pulmonary function in healthy blacks and whites. Respir Physiol 33: 367–393. 705072 10.1016/0034-5687(78)90063-4

[22] Cotes JE (1993) Lung function. 5th edition. Oxford: Blackwell Scientific Publications.

[23] Durnin JVGA, Womersley J 974 Body fat assessed from total body density and its estimation from skinfold thickness: measurements on 481 men and women aged from 16 to 72 years. Br J Nutr 32: 77–97. 4843734 10.1079/bjn19740060

[24] Cole TJ, Green PJ (1992) Smoothing reference centile curves: the LMS method and penalized likelihood. Stat Med 11: 1305–1319. 1518992 10.1002/sim.4780111005

[25] Cole TJ, Stanojevic S, Coates AL, Hankinson JL, Wade AM (2009) Age- and size related reference ranges: a case study of spirometry through childhood and adulthood. Stat Med 28: 880–898. 19065626 10.1002/sim.3504 PMC2798072

